# Heteroresistance to Fluconazole Is a Continuously Distributed Phenotype among *Candida glabrata* Clinical Strains Associated with *In Vivo* Persistence

**DOI:** 10.1128/mBio.00655-16

**Published:** 2016-08-02

**Authors:** Ronen Ben-Ami, Offer Zimmerman, Talya Finn, Sharon Amit, Anna Novikov, Noa Wertheimer, Mor Lurie-Weinberger, Judith Berman

**Affiliations:** a Infectious Diseases Unit, Tel Aviv Sourasky Medical Center, Tel Aviv, Israel; b Sackler School of Medicine, Tel Aviv, Israel; c Department of Molecular Microbiology and Biotechnology, George Wise Faculty of Life Sciences, Tel Aviv University, Tel Aviv, Israel

## Abstract

*Candida glabrata* causes persistent infections in patients treated with fluconazole and often acquires resistance following exposure to the drug. Here we found that clinical strains of *C. glabrata* exhibit cell-to-cell variation in drug response (heteroresistance). We used population analysis profiling (PAP) to assess fluconazole heteroresistance (FLC^HR^) and to ask if it is a binary trait or a continuous phenotype. Thirty (57.6%) of 52 fluconazole-sensitive clinical *C. glabrata* isolates met accepted dichotomous criteria for FLC^HR^. However, quantitative grading of FLC^HR^ by using the area under the PAP curve (AUC) revealed a continuous distribution across a wide range of values, suggesting that all isolates exhibit some degree of heteroresistance. The AUC correlated with rhodamine 6G efflux and was associated with upregulation of the *CDR1* and *PDH1* genes, encoding ATP-binding cassette (ABC) transmembrane transporters, implying that HetR populations exhibit higher levels of drug efflux. Highly FLC^HR^ *C. glabrata* was recovered more frequently than nonheteroresistant *C. glabrata* from hematogenously infected immunocompetent mice following treatment with high-dose fluconazole (45.8% versus 15%, *P* = 0.029). Phylogenetic analysis revealed some phenotypic clustering but also variations in FLC^HR^ within clonal groups, suggesting both genetic and epigenetic determinants of heteroresistance. Collectively, these results establish heteroresistance to fluconazole as a graded phenotype associated with ABC transporter upregulation and fluconazole efflux. Heteroresistance may explain the propensity of *C. glabrata* for persistent infection and the emergence of breakthrough resistance to fluconazole.

## IMPORTANCE

Heteroresistance refers to variability in the response to a drug within a clonal cell population. This phenomenon may have crucial importance for the way we look at antimicrobial resistance, as heteroresistant strains are not detected by standard laboratory susceptibility testing and may be associated with failure of antimicrobial therapy. We describe for the first time heteroresistance to fluconazole in *C. glabrata*, a finding that may explain the propensity of this pathogen to acquire resistance following exposure to fluconazole and to persist despite treatment. We found that, rather than being a binary all-or-none trait, heteroresistance was a continuously distributed phenotype associated with increased expression of genes that encode energy-dependent drug efflux transporters. Moreover, we show that heteroresistance is associated with failure of fluconazole to clear infection with *C. glabrata*. Together, these findings provide an empirical framework for determining and quantifying heteroresistance in *C. glabrata*.

## INTRODUCTION

*Candida glabrata* is an important opportunistic pathogen notable for its intrinsic tendency to develop resistance to antifungal drugs. Specifically, *C. glabrata* isolates are inhibited by higher concentrations of fluconazole and other azoles than most other *Candida* species (1) and are frequently resistant to this class of drugs (2, 3). Azole resistance is usually associated with upregulation of transmembrane ATP-binding cassette (ABC) drug transporters (4, 5). These characteristics have been the basis of recommendations to prefer echinocandins as the front-line treatment for *C. glabrata* and to use maximal doses of fluconazole for the treatment of apparently azole-sensitive isolates (6, 7). Importantly, echinocandin resistance is also emerging (8, 9), implying that the prospect of multidrug-resistant *C. glabrata* may soon become a reality (10).

A striking characteristic of *C. glabrata* is the ability of seemingly susceptible strains to become fully azole resistant during fluconazole treatment (4). The emergence of drug resistance during treatment implies the existence of nonsusceptible subpopulations within a predominantly susceptible isogenic microbial population. This phenomenon, known as heteroresistance (HR), has been described in Gram-positive and Gram-negative bacterial pathogens, in *Mycobacterium tuberculosis* (11–13), and in the pathogenic yeast *Cryptococcus neoformans* (14). When heteroresistant strains are serially cultured on media containing inhibitory concentrations of an antimicrobial drug, each generation exhibits variable drug responses but the nonsusceptible subpopulation gradually expands and fully resistant colonies will ultimately emerge. Population analysis profiling (PAP) assays are considered the gold standard for determining HR (13). In a wider context, HR is a manifestation of bet hedging, a mechanism whereby genetically identical cells express different phenotypic profiles, thus increasing the probability of survival during stressful fluctuations of environmental conditions (15). Potential implications of HR include treatment failure, relapse, and the establishment of persistent chronic infection (13). HR is generally not detected in standard broth microdilution susceptibility assays, and failure to detect HR may result in misclassification of nonsusceptible strains as susceptible by clinical microbiology laboratories. However, the lack of a standardized definition of HR complicates attempts to determine the clinical importance of this phenomenon (13).

We explored fluconazole HR (FLC^HR^) in *C. glabrata* by performing PAP of clinical isolates. We then tested the gene expression and function of ABC drug transporters in *C. glabrata* strains exhibiting different degrees of FLC^HR^. Finally, we determined the effect of HR on *in vivo* survival during fluconazole treatment. Our results demonstrate that fluconazole HR is a continuously distributed phenotype in *C. glabrata* clinical isolates. The area under the PAP curve provides a quantitative measurement of FLC^HR^ and correlates with ATP-dependent efflux transporter activity. Importantly, FLC^HR^ is not detected by routine susceptibility testing methods and could be an important factor underlying treatment failure.

## RESULTS

### Drug susceptibility population distribution analysis.

**FIG 1:**
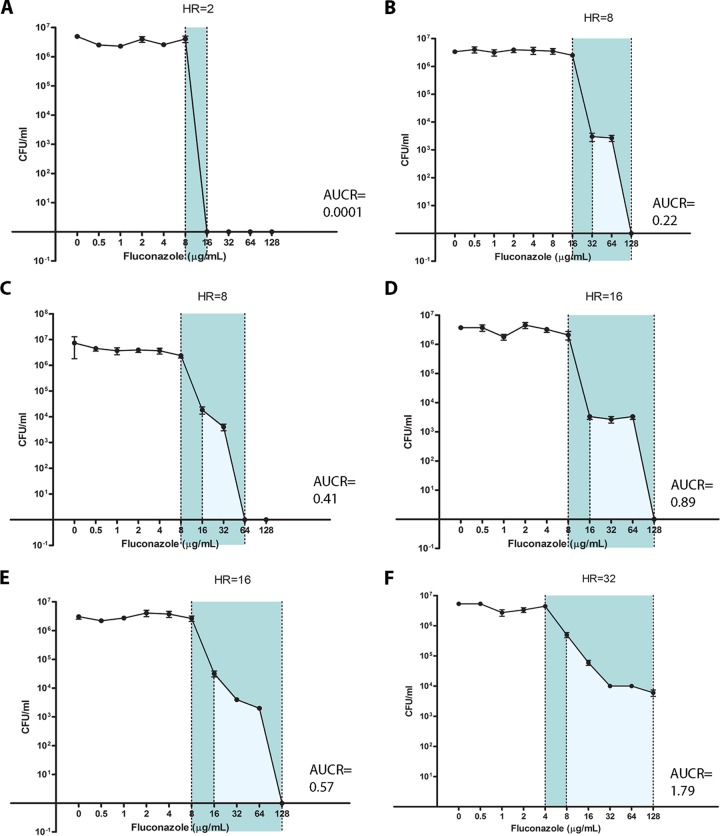
Fluconazole population analysis profiles define heteroresistance phenotypes. PAP curves for *C. glabrata* strains with various degrees of FLC^HR^ are shown (A to F). Population analysis patterns were obtained by fluconazole agar dilution as described in Materials and Methods. The heterogeneity range (HR) is marked in dark blue, and the area under the PAP curve (AUC) is marked in light blue. Note that the AUCRs differ widely between strains that share the same HR values (B and C, D and E). Each data point represents the mean ± the standard error of the mean.

**FIG 2:**
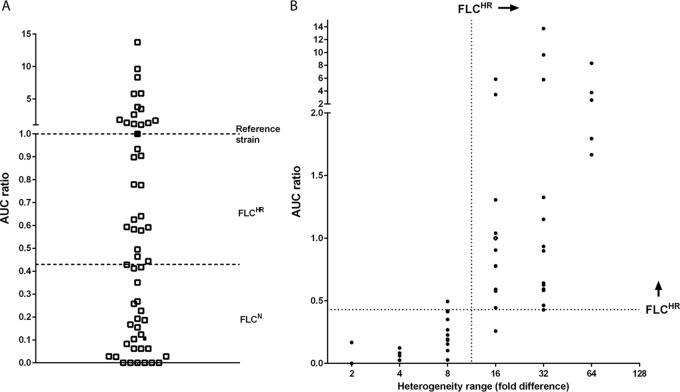
FLC^HR^ is distributed continuously among *C. glabrata* strains. The fluconazole AUCR distribution of 44 *C. glabrata* strains (A) shows a continuum of heteroresistance states over a wide range of values. Isolates Cg1775 and Cg1646, which were used in mouse infection studies, are represented by black squares. The correlation between the heterogeneity range and the AUCR (B) demonstrates that a dichotomous definition of FLC^HR^ based on an HR breakpoint of 16 correlates with an AUCR breakpoint of 0.43. However, the AUCR varies widely within each HR value category.

The fluconazole susceptibility characteristics of 52 *C. glabrata* clinical isolates and three reference strains (median fluconazole MIC of 2 mg/liter; range, 0.25 to 16 mg/liter; see Table S1 in the supplemental material) were determined by PAP. PAP assays were analyzed using the heterogeneity range (HR) method, which categorizes strains in a dichotomous fashion as fluconazole heteroresistant (FLC^HR^) or nonheteroresistant (FLC^N^), and by using the area under the curve (AUC) ratio (AUCR), which provides a continuous measurement of heterogeneity (Fig. 1). AUCRs ranged from 0.00012 to 13.74 (median 0.46; interquartile range, 0.10 to 1.12; Fig. 2A). Correlating the AUCR with fluconazole dose-response heterogeneity showed a partition between AUCRs for HRs of <16 and ≥16. Specifically, the median AUCR was 0.09 (range, 0.00012 to 0.41) for an HR of <16 and 0.96 (0.25 to 13.74) for an HR of ≥16 (*P* < 0.0001; Fig. 2B). An AUCR breakpoint of 0.43 categorized 28 (93%) of 30 strains with an HR of ≥16 as FLC^HR^ and 24 (96%) of 25 strains with an HR of <16 as FLC^N^. However, for each HR value of ≥16, the AUCRs varied up to 30-fold: 0.25 to 5.85 for an HR of 16, 0.42 to 13.74 for an HR of 32, and 1.66 to 8.32 for an HR of 64 (Fig. 2). This indicates that rather than being a bimodally distributed phenotype, FLC^HR^ is a continuously distributed trait in *C. glabrata*. In all, 30 (57.6%) of 52 clinical *C. glabrata* isolates were FLC^HR^ according to an HR breakpoint of ≥16. Population fluconazole susceptibility profiles were stable for each strain, with minimal variation in fluconazole AUCRs after >10 passages on fluconazole-free medium (data not shown). The fluconazole MIC was slightly but significantly higher for FLC^HR^ strains than for FLC^N^ strains (MIC_50_ [interquartile range], 2 [1 to 4] µg/ml versus 1 [1 to 2] µg/ml; *P* = 0.02).

**FIG 3:**
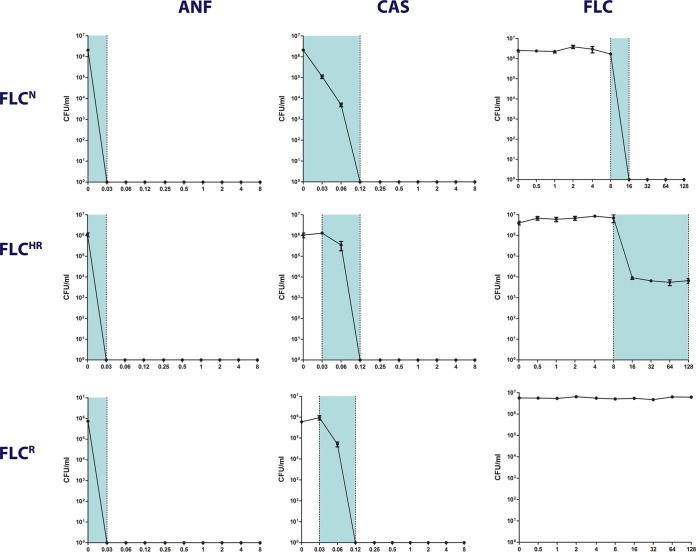
Differing PAP patterns of fluconazole, caspofungin, and anidulafungin. The PAP patterns of three representative *C. glabrata* strains are shown: fluconazole-susceptible nonheteroresistant (FLC^N^) reference strain CBS15126, FLC^HR^ clinical strain Cg1462, and fluconazole-resistant clinical strain Cg1708. ANF, anidulafungin; CAS, caspofungin; FLC, fluconazole. Each data point represents the mean ± the standard error of the mean.

To determine the prevalence of HR to echinocandins, we performed caspofungin and anidulafungin PAP assays of all of our *C. glabrata* isolates. All of the isolates tested had a heterogeneity range of 2 to 4 for echinocandins, indicating no significant HR to these drugs. There was no difference in echinocandin HR among isolates that were FLC^N^, FLC^HR^, and fluconazole resistant (Fig. 3). All of our *C. glabrata* isolates grew on 2% (wt/vol) glycerol as the sole carbon source, indicating the absence of mitochondrial mutations leading to respiration-defective (petite) mutants (16).

### Rhodamine 6G efflux and accumulation correlate with HR.

**FIG 4:**
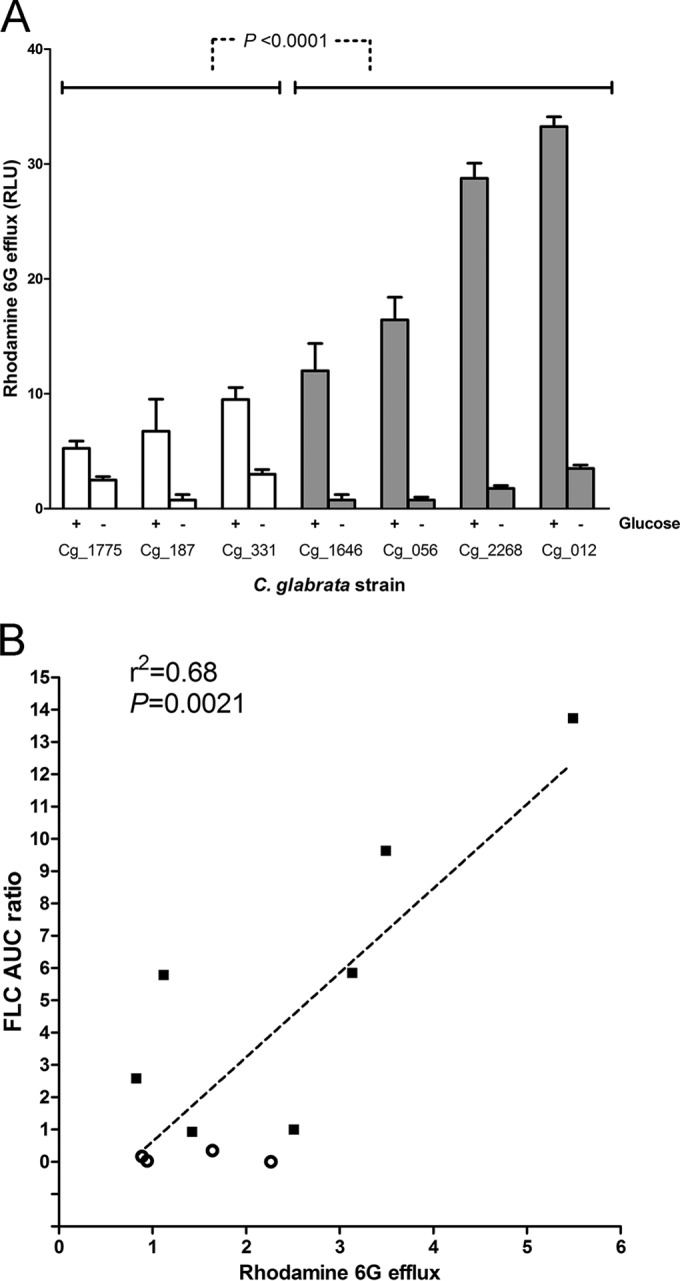
Rhodamine 6G efflux correlates with FLC^HR^. (A) Rhodamine 6G efflux was measured in the presence of 8 mM glucose (+ glucose) and under glucose starvation conditions (− glucose) to differentiate ABC transmembrane transporter specific activity. White bars, FLC^N^ strains; dark bars, FLC^HR^ strains. In aggregate, FLC^HR^ had significantly greater ABC-type efflux activity. Yet, as with the AUCR, rhodamine 6G efflux was distributed continuously rather than bimodally between FLC^HR^ and FLC^N^ strains. RLU, relative luminescence units. (B) Correlation between rhodamine 6G efflux activity and degree of FLC^HR^, expressed as FLC-AUCR. Empty circles, FLC^N^ strains; black squares, FLC^HR^ strains.

We measured the glucose-dependent efflux activity of ABC-type transporters in representative FLC^HR^ and FLC^N^ *C. glabrata* strains with the rhodamine 6G assay (17). As expected of ABC transporters, rhodamine 6G efflux, determined by relative fluorescence in supernatants, occurred rapidly after the addition of glucose to yeast cell suspensions and was absent from glucose-free controls (Fig. 4). Glucose-dependent rhodamine 6G efflux was significantly higher in FLC^HR^ strains than in FLC^N^ strains (*P* < 0.001; Fig. 4). However, rhodamine 6G efflux activity varied markedly within the FLC^N^ and FLC^HR^ categories, with no clear breakpoint between FLC^N^ and FLC^HR^ strains, as defined by heterogeneity range criteria. Moreover, rhodamine 6G efflux correlated positively and linearly with the degree of FLC^HR^, expressed as the AUCR (*r*^2^ = 0.68, *P* = 0.0021; Fig. 4).

**FIG 5:**
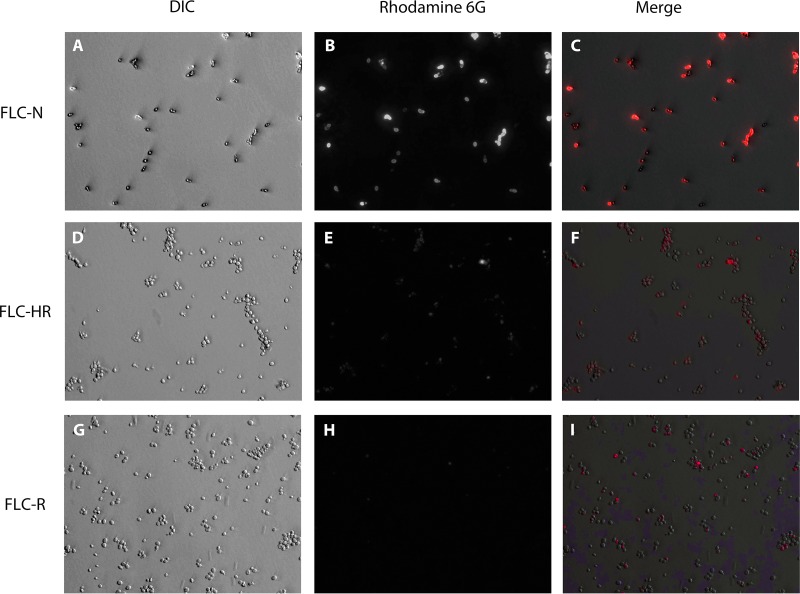
Visualization of intracellular rhodamine 6G accumulation. Rhodamine 6G accumulation in cells was detected by fluorescence microscopy. Panels: A to C, FLC^N^ isolate Cg1775; D to F, FLC^HR^ isolate Cg2268; G and H, FLC^R^ isolate Cg1708; A, D, and G, light microscopy; B, E, and H, fluorescence microscopy (rhodamine filter); C, F, and I, composite images. All images were captured at ×400 magnification by using manually fixed exposure values. DIC, differential interference contrast.

Evaluation of rhodamine 6G retention in yeast cells by fluorescence microscopy allows visual assessment of efflux activity within a cell population. There was cell-to-cell variation in rhodamine 6G accumulation within both FLC^HR^ and FLC^N^ populations (Fig. 5). However, the proportion of cells stained with rhodamine 6G following a 25-min incubation in phosphate-buffered saline (PBS) with 8 mM glucose was lower for the FLC^HR^ strain than for the FLC^N^ strain. Accumulation of rhodamine 6G was almost absent from FLC^R^ cells and was lower than that in both FLC^HR^ and FLC^N^ strains (Fig. 5).

Taken together, these results indicate that the fluconazole AUCR of a given *C. glabrata* strain reflects the overall ABC transporter efflux activity of the cell population, which in turn is an expression of the proportion of cells with increased efflux activity.

### Individual ABC transporter genes are upregulated in a binary manner in FLC^HR^ strains.

**FIG 6:**
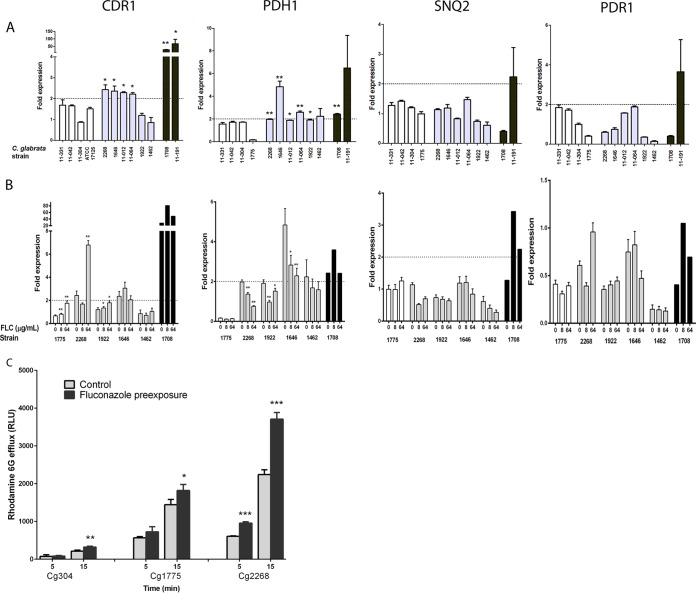
Expression of genes encoding efflux transporters. (A) Relative expression of efflux transporter genes *CDR1*, *PDH1*, and *SNQ2* and transcription factor *PDR1* in four FLC^N^ isolates (white bars), six FLC^HR^ isolates (gray bars), and two FLC^R^ isolates (black bars). Each dashed line marks the arbitrary 2.0-fold threshold of upregulation. *, *P* < 0.05; **, *P* < 0.01 (for the comparison of gene expression with the mean expression of four FLC^N^ isolates). Note that the *y* axis is on a different scale for each gene. (B) Induction of efflux transport-associated genes following a 3.5-h incubation in drug-free medium or medium containing fluconazole (FLC) at 8 or 64 µg/ml. *, *P* < 0.05; **, *P* < 0.01 (for relative gene expression induction following drug exposure). Expression was normalized to *ACT1* and then compared to levels of expression in the same strain that was not exposed to the drug. *PDR1* expression never reached the 2-fold threshold for upregulation. Each data point represents the mean ± the standard deviation of three experiments. (C) Rhodamine 6G efflux of *C. glabrata* strains Cg304, Cg1775 (FLC^N^), and Cg2268 (FLC^HR^) after incubation with fluconazole (8 µg/ml, black bars) or a saline control (gray bars). *, *P* < 0.05; **, *P* < 0.01; ***, *P* < 0.0001.

We compared the ACT1-normalized expression of the three azole efflux transporter genes (*CDR1*, *PDH1*, *SNQ2*) and the ABC transport regulator gene *PDR1* among FLC^N^ (*n* = 4), FLC^HR^ (*n* = 6), and FLC^R^ (*n* = 2) *C. glabrata* strains (Fig. 6). As expected, in FLC^R^ strains, *CDR1* expression was dramatically higher than in both FLC^N^ and FLC^HR^ strains (mean expression, 47.6-fold ± 19.8-fold; *P* < 0.0001) (Fig. 6). Modest but significant increases in *CDR1* expression levels were observed in four of six FLC^HR^ strains, whereas none of four FLC^N^ strains had increased *CDR1* expression (mean expression, 2.32-fold ± 0.04-fold and 1.43-fold ± 0.19-fold, respectively; *P* = 0.028). In addition, *PDH1* expression was significantly increased in five of six FLC^HR^ strains versus none of the FLC^N^ strains (mean expression, 2.63-fold ± 0.56-fold versus 1.29-fold ± 0.37-fold; *P* = 0.015). In sum, four of six FLC^HR^ strains upregulated both *CDR1* and *PDH1* and an additional FLC^HR^ strain upregulated *PDH1* alone (Fig. 6). In contrast, there was no significant difference in the expression of *SNQ2* and *PDR1* between FLC^N^ and FLC^HR^ strains, suggesting that transporters other than Cdr1 and Pdh1 (Snq2 and the Pdr1 transcription factor) do not play a major role in FLC^HR^.

Unlike fluconazole efflux, expression of *CDR1* and *PDH1* was not linearly related to the fluconazole AUCR. The fold expression values of both genes were distributed bimodally among FLC^HR^ and FLC^N^ strains, suggesting dichotomous “on-off” states of gene expression (see Fig. S1 in the supplemental material). Both FLC^HR^ strains that did not upregulate *CDR1* had low rhodamine 6G efflux activity (see Fig. S1). Upregulation of *CDR1* and *PDH1* was associated with high values of rhodamine 6G efflux, but the difference from that in strains not upregulating these genes did not reach statistical significance (*P* = 0.1; see Fig. S1).

Preexposure to fluconazole (8 or 64 mg/liter) resulted in significant upregulation of *CDR1* expression in two FLC^HR^ strains: Cg2268, which upregulated *CDR1* at the baseline (2.8-fold increase in *CDR1* expression following preexposure; *P* < 0.0001), and Cg1922, which did not upregulate *CDR1* at the baseline (50% increase in expression; *P* = 0.008; Fig. 6). There was no significant effect of fluconazole exposure on the *CDR1* expression of two other FLC^HR^ strains. In contrast, *PDH1* expression in all FLC^HR^ strains preexposed to fluconazole was reduced to 38 to 70% of the baseline values (Fig. 6). The expression levels of both *SNQ2* and *PDR1* following preexposure to fluconazole remained well below the 2-fold threshold for all FLC^HR^ strains, indicating that *CDR1* upregulation in FLC^HR^ strains is not mediated through altered Pdr1 levels (18). Rhodamine 6G efflux increased in all *C. glabrata* strains after incubation with fluconazole, but the increase was more rapid and pronounced in strain Cg2268 than in FLC^N^ strains Cg304 and Cg1775 (65% increase [*P* <0.0001] versus 26 to 51% increase [*P* = 0.01]; Fig. 6C). Thus, upregulation of *CDR1* by fluconazole preexposure was associated with enhanced rhodamine 6G efflux.

Sequencing of *PDR1* in six FLC^HR^ strains and four FLC^N^ strains revealed seven nonsynonymous mutations in the coding sequence (see Table S2 in the supplemental material). *PDR1* mutations were detected in three of six FLC^HR^ strains and two of four FLC^N^ strains. These mutations were not associated with a gain-of-function phenotype and did not corresponded to previously described *PDR1* gain-of-function substitutions (19–21).

### HR is associated with persistence of viable *C. glabrata* cells in mouse kidney tissue during fluconazole treatment.

**FIG 7:**
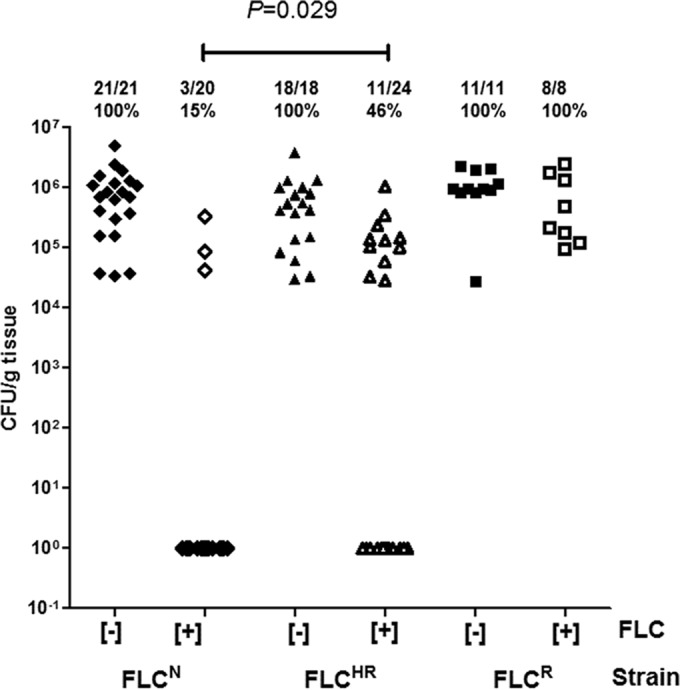
Persistence of viable *C. glabrata* in kidney tissue despite fluconazole treatment. Persistence of viable *C. glabrata* in homogenized kidneys of immunocompetent BALB/c mice was determined 7 days after tail vein infection. Diamonds, Cg1775 (FLC^N^ isolate); triangles, Cg1646 (FLC^HR^ isolate); squares, Cg1708 (FLC^R^ isolate); empty symbols, mice injected intraperitoneally with fluconazole 100 mg/kg/day; full symbols, mice treated with saline control. Values at the top are the numbers of mice with persistence of viable *C. glabrata* in the kidneys at the end of 7 days of fluconazole treatment, the total number of mice in each group, and the percentage with persistent infection.

To determine whether FLC^HR^ correlates with failure of fluconazole to eliminate *C. glabrata* from visceral tissue, we assessed the viable fungal burdens (in numbers of CFU per gram of tissue) in mouse kidneys 7 days after intravenous inoculation. High-dose fluconazole treatment (100 mg/kg/day) significantly reduced the tissue fungal burdens of FLC^N^ and FLC^HR^ *C. glabrata*-infected mice (*P* < 0.0001) and, as expected, had no effect on FLC^R^ *C. glabrata*-infected mouse tissue fungal burdens (Fig. 7). Individual mice with persistent *C. glabrata* infection after 7 days of fluconazole treatment could be detected in both the FLC^N^ and FLC^HR^ infection groups. Persistent infection manifested itself as high tissue fungal burdens approaching those observed in untreated mice (2.0 × 10^5^ ± 7 × 10^4^ and 8.6 × 10^5^ ± 1.6 × 10^5^ CFU/g, respectively; Fig. 7). Importantly, the frequency with which persistent infection was detected was higher in fluconazole-treated mice infected with a highly FLC^HR^ strain (11 of 24 mice, 45.8%) than in fluconazole-treated mice infected with an FLC^N^ strain (3 of 20 mice, 15%) (*P* = 0.029), despite both strains having fluconazole MICs in the susceptible range.

### FLC^HR^ is variable within phylogenetic clusters.

**FIG 8:**
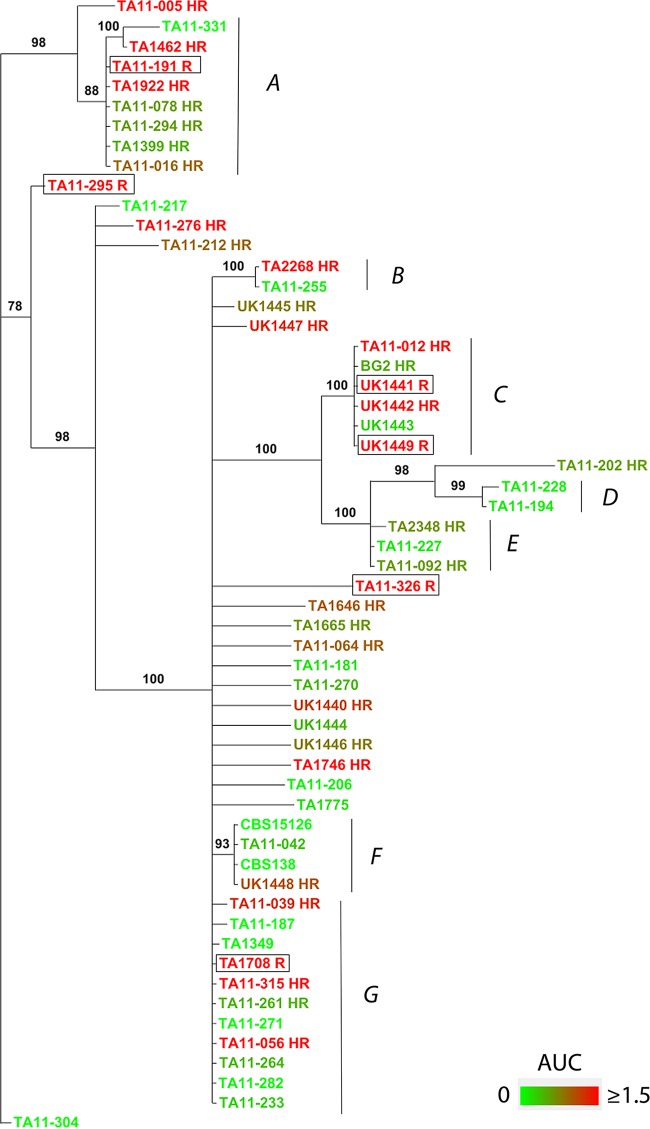
Phylogenetic distances among strains with different fluconazole susceptibility phenotypes. Phylogenetic relationships were derived by comparing the sequences of the IGS region between nuclear genes *CDH1* (CAGL0A00605g) and *ERP6* (CAGL0A00627g) of *C. glabrata* strains. The cladogram was constructed by MCMC methodology. Posterior probabilities are shown at the nodes. The color gradient correlates with the degrees of FLC^HR^ (AUCR legend). Capital letters mark the main clusters referred to in the text. Fluconazole-resistant strains are framed.

Comparison of the PAP phenotypes and phylogenetic relationships determined from intergenic spacer (IGS) sequences showed clusters of *C. glabrata* strains with similar phenotypes, as well as variable AUCRs, among closely related strains (Fig. 8). Two clusters (A and C) were composed of closely related FLC^HR^ strains. Interestingly, both clusters also contained FLC^R^ strains (one in cluster A and two in cluster C). The six fully fluconazole-resistant strains included in this analysis did not cluster with each other; four of the FLC^R^ strains were found within clusters shared by FLC^HR^ strains, and two strains were singletons (Fig. 8).

Population heat mapping of *C. glabrata* strains according to AUCRs showed that closely related *C. glabrata* strains often have divergent AUCRs (clusters A, B, C, F, and G; Fig. 8). In sum, phylogenetic analysis shows both phenotypic clustering and dynamic changes in the fluconazole susceptibility patterns of related *C. glabrata* strains.

## DISCUSSION

Stochastic cell-to-cell variation in gene expression within genetically identical populations is a postulated mechanism for microbial survival under fluctuating environmental conditions, also referred to as bet hedging (22). HR is a specific manifestation of bet hedging as it pertains to antimicrobial resistance (11–13, 23). However, fundamental questions remain regarding HR. First, a uniform definition of HR has not been agreed upon (13). More importantly, it remains unclear whether HR is a binary characteristic or a continuously distributed phenotype and whether similar definitions should apply to prokaryotic bacteria and eukaryotic fungi (15). Second, the molecular underpinnings of HR remain unclear. Finally, the importance of HR as a mechanism of microbial persistence and treatment failure *in vivo* has yet to be determined (11–13). Here, we show that FLC^HR^ is a continuously distributed phenotype in *C. glabrata*, a major human fungal pathogen that excels in the ability to become fully resistant following exposure to this drug (4). Quantitative measurement of HR by using the fluconazole AUC of the population analysis plot (AUCR) provided an indirect assessment of the proportion of resistant cells within the total population of each strain, as evidenced by the linear correlation between the AUCR and ABC-type efflux activity. Importantly, *in vivo* persistence during treatment with high-dose fluconazole was significantly associated with FLC^HR^, providing evidence that HR may be a cause of treatment failure and relapse observed in the clinical setting (24).

Using a binary definition of HR suggested in a recent review (13, 25), 57.6% (30/52) of the fluconazole-susceptible *C. glabrata* isolates included in this study met the criteria for FLC^HR^. However, the continuous distribution of the AUCR among FLC^HR^ and FLC^N^ strains (Fig. 2) and the correlation of this index with rhodamine 6G efflux indicate that FLC^HR^ is more appropriately treated as a graded phenotype. This observation is consistent with the work of Levy et al. (15), who described a continuum of growth rates within the cell population of *Saccharomyces cerevisiae*, a nonpathogenic yeast closely related to *C. glabrata*. Those researchers hypothesized that in yeast, a combination of stochastic and deterministic factors dictate a spectrum of inheritable epigenetic states that confer different levels of fitness (15). In contrast, a bimodal distribution of persister and nonpersister cells has been described in bacterial populations (22). Our findings extend the graded nature of yeast cell states to the interstrain population level.

In the majority of FLC^HR^ isolates, we detected a low level of ABC transmembrane transporter gene *CDR1* and *PDH1* upregulation (4). These expression levels were much lower than those seen in FLC^R^ isolates (i.e., ~2-fold versus 50-fold), likely because in a heteroresistant population, only about 1 in 10^3^ cells exhibits a resistance phenotype, thus diluting the observed increase in transporter transcripts. Moreover, none of the FLC^HR^ *C. glabrata* isolates showed evidence of a gain-of-function mutation in *PDR1*, which has been reported to cause full resistance to fluconazole (19). Consistent with this, no significant increase in *SNQ1* expression, whose transcription is also regulated by Pdr1p, was detected. In contrast to rhodamine 6G efflux, *CDR1* and *PDH1* expression was increased to similar levels in FLC^HR^ strains, irrespective of their AUCRs (Fig. 7). Thus, the FLC^HR^ phenotype seems to represent the incremental effects of multiple binary genetic switches, whose sum produces a spectrum of HR states.

The clinical significance of HR remains largely unclear for the bacteria in which it has been described (11). Here, we determined the correlation between high-level HR and persistent infection during a simulated course of high-dose fluconazole treatment by using a mouse model of hematogenously disseminated candidiasis. A significantly larger proportion of mice infected with an FLC^HR^ isolate than of mice infected with an FLC^N^ strain had high kidney fungal burdens after 7 days of fluconazole treatment. Interestingly, some of the FLC^HR^ strains did not show a kidney fungal burden at day 7 and a kidney fungal burden was observed in mice infected with the FLC^N^ strain, albeit less frequently. This finding is consistent with our *in vitro* results and supports the notion that some degree of HR is present in most *C. glabrata* isolates.

Internal diversity in population drug susceptibility may arise from genetic, epigenetic, or nongenetic mechanisms. We analyzed genetic relatedness to gain insight into the evolution and dynamics of the FLC^HR^ phenotype. We found phenotypic clustering of some FLC^HR^ and FLC^N^ strains, as well as significant variability of AUCRs among some genetically related *C. glabrata* isolates. These relationships suggest that FLC^HR^ represents an interplay of genetic and epigenetic mechanisms (Fig. 8).

Resistance of *C. glabrata* to fluconazole varies widely between isolates from different geographical regions, as well as within those regions (2, 3). We found high rates of FLC^HR^ among *C. glabrata* clinical isolates collected in Israel and the United Kingdom. Together with our phylogenetic data, this geographic pattern is consistent with the circulation of multiple *C. glabrata* clades with different propensities to evolve full azole resistance. The degree to which regional differences in azole use drives this process requires further study.

Given the epidemiological and clinical importance of *C. glabrata*, we suggest that HR may be a driver of azole resistance, treatment failure, and unresolved infection. Of note, surveillance efforts have focused solely on azole resistance and do not account for the HR phenotype, which may be much more prevalent. This discrepancy may explain the inconsistent correlation observed between fluconazole MICs and clinical outcomes of treating *C. glabrata* infection with this drug (26). Interestingly, we did not detect HR to echinocandins in any of the clinical *C. glabrata* isolates, consistent with the better correlation between echinocandin MICs and the responses to treatment (8, 27).

It has been postulated that because of the fungistatic nature of azoles, *Candida* cells exposed to these drugs enter an arrested state in which stress-induced genetic instability promotes the development of drug resistance and breakthrough infection (28). In this context, enhanced *in vivo* persistence of FLC^HR^ *C. glabrata* during fluconazole treatment, as observed in the mouse model, creates optimal conditions for the emergence of stress-induced mutations conferring azole resistance. The lack of significant HR to echinocandins may be related to the rapid fungicidal effect of glucan-synthase inhibition, which does not allow time for stress-induced genetic instability. It should be noted, however, that of the 52 clinical *C. glabrata* strains in this study, only 8 were recovered from patients treated with an azole during the preceding year (see Table S1 in the supplemental material). Hence, other types of environmental stress were probably involved in the generation of FLC^HR^ strains. ABC transporters have broad substrate specificity, allowing them to export a wide range of cytotoxic molecules, including organic acids, sterols, and H^+^ ions (29). FLC^HR^ may thus represent part of a more general stress-induced phenotype associated with the upregulated expression of ABC transporter genes.

Mechanisms of HR remain poorly understood at the molecular and single-cell levels (23). Our findings establish that FLC^HR^ appears to be frequent in clinical *C. glabrata* isolates, affecting more than half of the strains tested. Quantitative PAP analysis revealed a continuous range of FLC^HR^ rather than a categorical phenotype, suggesting that multiple determinants are involved. Additional studies should be undertaken to identify the range of genetic and/or physiological determinants that contribute to FLC^HR^ and to elucidate the significance of the FLC^HR^ phenotype in clinical practice.

## MATERIALS AND METHODS

### Ethics statement.

All animal experiments were approved by the Tel Aviv Sourasky Medical Center (TASMC) Institutional Animal Care and Use Ethics Committee (IACUC) under protocol b3703-11-037 and conform to the Israeli Animal Experimentation Law and U.S. Public Health Service Policy on Humane Care and Use of Laboratory Animals. Clinical data extraction was approved by the TASMC Ethics Committee under protocol 0217-11-TLV in adherence to the Israeli Ministry of Health guidelines for clinical trials with human subjects.

### *Candida* strains.

*C. glabrata* strains were obtained from clinical samples collected at the TASMC microbiology laboratory (48 strains) and at the University of Exeter, United Kingdom (10 strains). Each strain was specific to a single patient. Strains were identified to the species level with the Vitek 2 system and the YST ID card (bioMérieux, Marcy l’Etoile, France) and by sequencing of internal transcribed spacer (ITS) regions ITS1 and ITS2 (30). All of our *C. glabrata* isolates (38 bloodstream and 14 mucosal isolates) were determined to be susceptible to fluconazole, voriconazole, and itraconazole by broth microdilution according to CLSI methods outlined in document M27-A3 (31). *C. glabrata* strains CBS15126, CBS138, and BG2 were used as fluconazole-susceptible reference strains. *C. glabrata* clinical isolates Cg1708, Cg191, and Cg326 served as fluconazole-resistant controls.

### Drug susceptibility PAP.

Although there are no formal standards for determining HR, PAP is generally considered the most robust method (13). We analyzed the population distribution of *Candida* isolates for susceptibility to fluconazole, caspofungin, and anidulafungin by a plating dilution method (32). Yeast extract agar glucose (YAG; yeast extract at 5 g/liter; glucose at 10 g/liter; agar at 15 g/liter; 1 M MgCl_2_ at 10 ml/liter; trace elements at 1 ml/liter) was prepared with nine different concentrations of fluconazole (log_2_ serial dilutions ranging from 128 to 0.5 mg/liter), caspofungin (8 to 0.03 mg/liter), and anidulafungin (8 to 0.03 mg/liter). Twenty milliliters of each YAG-drug concentration or a drug-free YAG control was poured into 20-cm culture plates and allowed to cool. *Candida* strains were passaged once on Sabouraud’s agar to ensure purity. Two or three colonies were suspended in sterile saline, and hemocytometer counts were used to adjust the density to 10^6^ yeast cells/ml. Four log_10_ dilutions of yeast cell suspension were prepared, corresponding to 10^5^ to 10^2^ CFU/ml. Each YAG-drug plate was divided into quadrants, and each quadrant was inoculated with six 5-µl drops of a different yeast cell suspension concentration and marked accordingly. Plates were incubated at 30°C for 24 h, and the optimal yeast concentration at which nonconfluent colonies could be enumerated was used to count CFU and to calculate the number of viable cells per milliliter. For each *Candida* strain, the viable cell density (in CFU per milliliter) was plotted over the drug concentration (13). To quantitate FLC^HR^, we calculated the area under the population distribution curve (AUC) (32). In order to increase the sensitivity of this method to detect variations in *Candida* growth at high fluconazole concentrations, we analyzed the AUCs for fluconazole concentrations ranging from the MIC to 128 mg/liter (Fig. 1). To enhance robustness across different experiments, the AUC was normalized by dividing it by the same value for a well-characterized FLC^HR^ *C. glabrata* strain (Cg1646), yielding an AUCR.

Bacterial HR is commonly defined in binary terms as a minimal fold difference between the lowest drug concentration associated with maximal growth inhibition and the highest noninhibitory concentration (25), referred to here as the heterogeneity range (Fig. 1). To determine a meaningful fold difference breakpoint for HR, we analyzed the correlation between the AUCR and the heterogeneity of drug response on PAP.

### Rhodamine 6G efflux and accumulation analyses.

To assess ABC-type drug transporter activity, we determined the glucose-induced efflux and cellular retention of rhodamine 6G as described previously (17, 33). *C. glabrata* isolates were grown to log phase in liquid YAG at 35°C. Yeast cells were collected by centrifugation, and 10^9^ cells were transferred to 20 ml of fresh YAG and incubated at 27°C for an additional 2 h. For drug exposure experiments, cells were incubated in PBS with fluconazole at 8 µg/ml or in drug-free PBS at 30°C in a shaking incubator for 3 h. Next, yeast cells were collected by centrifugation and washed twice in PBS, and 10 ml of PBS containing 15 µM rhodamine 6G without glucose was added to the pellets. Suspensions were vortexed and incubated at 27°C for 90 min to allow rhodamine 6G uptake under carbon source depleted conditions. Cells were collected by centrifugation, washed twice in PBS, and suspended in 750 µl of PBS in microcentrifuge tubes. To start rhodamine 6G efflux, 250 µl of PBS with 8 mM glucose of was added. Control tubes were prepared with glucose-free PBS. Tubes were removed after 5, 15, and 25 min of incubation at 35°C, and fluorescence was measured in 200-µl aliquots of supernatant with a spectrophotometer at an excitation wavelength of 527 nm and an emission wavelength of 555 nm (Synergy HT; BioTek, Winooski, VT). ABC-specific rhodamine efflux was calculated for each strain by subtracting the fluorescence in glucose-free supernatants from that in glucose-containing supernatants. To enhance interexperimental reproducibility, the rhodamine efflux of each strain was normalized to that of *C. glabrata* CBS15126, which was tested in each experiment. In separate experiments, the cellular accumulation of rhodamine 6G in *C. glabrata* strains was determined by fluorescence microscopy. Rhodamine 6G uptake and efflux were achieved as described above. After a 15-min incubation, suspensions were centrifuged and pellets were washed twice in PBS and observed under a triple-band fluorescence microscope (Olympus BX-51; Olympus, Melville, NY) with the rhodamine filter. Images were acquired with an Olympus DP71 camera (Olympus, Tokyo, Japan) and Cell^A software with the exposure kept constant at 74 ms.

### ABC transporter gene expression.

We assessed the association of the PAP phenotype with the expression of efflux transporter genes known to be associated with azole resistance in *C. glabrata* by real-time reverse transcription-quantitative PCR (RT-qPCR) as previously described (5). Isolates were grown in liquid YAG at 35°C in a shaking incubator to mid-exponential phase. Yeast cells were enumerated in a hemocytometer and transferred into fresh YAG with fluconazole at 8 or 64 mg/liter or into drug-free YAG at a final concentration of 10^7^/ml. Yeast suspensions were incubated for 3.5 h at 35°C with shaking; this stage did not affect *C. glabrata* viability, as determined by quantitative subculture on Sabouraud’s agar. Next, cells were pelleted, snap-frozen in liquid nitrogen, and thoroughly ground with 3-mm glass beads. Lysis buffer (Qiagen, Hilden, Germany) was added, and the lysate was further homogenized in a TissueLyser (Qiagen). RNA was extracted with the RNeasy kit (Qiagen) according to the manufacturer’s instructions, including in-column DNase treatment. cDNA was generated from 50 to 100 ng of total RNA with the High Capacity RNA-to-cDNA kit (Applied Biosystems/Life Technologies, Grand Island, NY). cDNA was diluted 1:10 in RNase/DNase-free water and subjected to RT-qPCR with primers and TaqMan probes for *CDR1*, *SNQ2*, *PDH1*, and *PDR1* (see Table S1 in the supplemental material). Each 25-µl reaction mixture included 5 µl of a cDNA template, 12.5 µl of ABI universal master mix, 0.4 µM each primer, and 0.2 µM TaqMan probe. PCRs were run in an ABI Prism 7500 sequence detection system (Applied Biosystems/Life Technologies) under the following conditions: 95°C for 10 min (AmpliTaq gold activation), followed by 40 cycles of 95°C for 15 s (denaturation) and 60°C for 60 s (annealing and extension). The amplification efficiency was determined for each gene. For each target gene, the mean threshold cycle (*C_T_*) was calculated from triplicate experiments and normalized to *ACT1* expression (Δ*C_T_*). Each gene was calibrated to the *ACT1*-normalized expression of its counterpart in nonheteroresistant *C. glabrata* strains CBS 15126 and Cg1775 (ΔΔ*C_T_*), and fold expression was calculated as 2^−ΔΔ*CT*^. A 2-fold increase in expression was arbitrarily set as the threshold for significant upregulation (5). The *PDR1* gene sequences of six FLC^HR^ and four FLC^N^ *C. glabrata* strains were analyzed (see Text S1 in the supplemental material).

### Persistent infection in a mouse model of hematogenous disseminated candidiasis.

*C. glabrata* is nonlethal when injected intravenously into immunocompetent mice; nonetheless, it can persist for prolonged periods in visceral tissue (34). To determine if the PAP phenotype is associated with persistent infection during fluconazole treatment *in vivo*, we compared the tissue burdens of Cg1775 (FLC^N^, fluconazole MIC of 0.25 mg/liter) and Cg1646 (FLC^HR^, fluconazole MIC of 1 mg/liter) in the kidneys of fluconazole-treated and untreated BALB/c mice. Cg1708 was used as a reference fluconazole-resistant strain. Eight-week-old female BALB/c mice weighing 20 to 25 g (Harlan, Rehovot, Israel) were infected by tail vein injection of 4 × 10^7^ cells in groups of 10 per *C. glabrata* strain. Five mice per strain were treated with fluconazole (Pfizer, New York) at 100 mg/kg once a day intraperitoneally. The remaining five mice were injected daily with a similar volume of sterile saline. Treatment was continued for 7 days postinfection, after which mice were sacrificed by CO_2_ inhalation and their kidneys were harvested and processed for quantitative determination of fungal burdens. Organs were weighed and homogenized in 1 ml of sterile saline with a TissueLyser set to 1 min at 50 Hz. Homogenates were serially diluted 10-fold and inoculated onto YAG plates for 48 h at 30°C. Fungal burdens were calculated and expressed as numbers of CFU per gram of tissue. Experiments were repeated three times.

### Phylogenetic analysis.

Phylogenetic relationships between different *C. glabrata* strains were resolved by sequencing and analysis of the rapidly evolving IGS region between nuclear genes *CDH1* and *ERP6* on chromosome A as described previously (35). Phylogenetic analysis included 48 fluconazole-susceptible *C. glabrata* isolates, 6 fluconazole-resistant clinical isolates, and reference *C. glabrata* strains CBS138, CBS15126, and BG2. After genomic DNA extraction, an ~660-bp fragment of the IGS was amplified with primers 00605 (5′ C TCA CAA ATG GAT TCC TTA AAG AGT TCG 3′) and 00627 (5′ GT C ACC AGA GTT GGA GTA CAT GTA G 3′). PCR was performed with 50-µl tubes containing 400 nM each primer under the following conditions: initial denaturation at 95°C (4.5 min) and then 35 cycles of 95°C (45 s), 52°C (1 min), and 72°C (1 min), followed by 72°C (7 min). The PCR products were sequenced with primers 00605 and 00627. Sequences were aligned with Clustal Omega (36), and phylogenetic trees were constructed in MrBayes 3.2 (37) by Bayesian Markov chain Monte Carlo (MCMC) methodology. The average standard deviation of split frequencies (ASDSF) was calculated by comparing split and clade frequencies across 1 × 10^6^ MCMC runs started from different randomly chosen trees. The ASDSF cutoff was set at <0.05. The final output is presented as a cladogram with the posterior probabilities for all of the splits.

### Statistics.

AUCRs of FLC^HR^ and FLC^N^ strains were compared with the Mann-Whitney test. Comparison of transporter gene transcript and rhodamine 6G fluorescence values was done by one-sided analysis of variance with the Bonferroni *post hoc* test to compare specific pairs of values. Correlations among HR, transporter gene expression, and rhodamine 6G efflux were explored by using linear regression. Fungal persistence in mice infected with *C. glabrata* was observed as a dichotomous outcome. Therefore, we compared groups of mice infected with different *C. glabrata* strains by considering fungal clearance a categorical variable. A two-sided type I error of 0.05 was used as the threshold for statistical significance. Calculations were performed in Prism 6.0 (GraphPad, San Diego, CA) and Stata 11.2 (StataCorp, College Station, TX).

### Data availability.

Representative *C. glabrata* strains (10 FLC^HR^ and 9 FLC^N^) have been deposited at the CBS-KNAW Fungal Biodiversity Centre, Utrecht, Netherlands (http://www.cbs.knaw.nl; see Table S1 in the supplemental material). The GenBank accession numbers of the CDH1-ERP6 IGS sequences used for phylogenetic analyses are listed in Table S1. The GenBank accession numbers of the *PDR1* gene sequences are listed in Table S2.

## SUPPLEMENTAL MATERIAL

Text S1  Supplemental methods used for *PDR1* gene sequence analysis. Download Text S1, DOCX file, 0.01 MB

Figure S1  Expression of *CDR1* and *PDH1* is distributed bimodally and correlates with FLC^HR^. Scatter plots show fold expression of *CDR1* (A and C) and *PDH1* (B and D) as a function of the fluconazole AUCR (A and B) and rhodamine 6G efflux (C and D). Empty circles, FLC^N^ strains; black squares, FLC^HR^ strains. The horizontal dashed line crosses the *y* axis at 2 for *CDR1* and 1.8 for *PDH1*. The graph describing *CDR1* expression as a function of rhodamine 6G efflux was fitted with the equation *y* = *y*_min_ + [(*y*_max_ − *y*_min_)/(1 + 10^logEC50 − *x*^)], where *y*_max_. and *y*_min_ are the maximal and minimal gene expression values and EC_50_ is the value of *x* at the midpoint of the slope. Download Figure S1, TIF file, 0.3 MB

Table S1  Clinical and reference *C. glabrata* strains used in this work. FLC, fluconazole; ITZ, itraconazole; VRC, voriconazole (see Materials and Methods); N, fluconazole nonheteroresistant; R, fluconazole resistant; NA, not applicable. An asterisk indicates treatment with fluconazole within 1 year prior to the isolation of *C. glabrata* Table S1, DOCX file, 0.02 MB

Table S2  Nonsynonymous mutations detected in the *PDR1* gene coding sequences of FLC^HR^ and nonheteroresistant *C. glabrata* strains. Table S2, DOCX file, 0.01 MB
