# Supplementary material for: Heteroresistance to Fluconazole Is a Continuously Distributed Phenotype among Candida glabrata Clinical Strains Associated with In Vivo Persistence
Source: mBio. 2016 Aug 2;7(4):e00655-16. doi: 10.1128/mBio.00655-16 (PMC4981708; doi:10.1128/mBio.00655-16)
Supplement: Text S1 — Supplemental methods used for PDR1 gene sequence analysis. Download [file mbo004162900s1.docx]

**Supplemental Methods**

The *PDR1* gene sequences of 6 FLC^HR^ and 4 FLC^N^ *C. glabrata* strains were analyzed from paired-end Illumina HiSeq runs (100 bp) by aligning raw reads with Bowtie2 (<http://bowtie-bio.sourceforge.net/bowtie2/index.shtml>) and sorting the relevant reads using SAMtools (<http://samtools.sourceforge.net/>). SNPs were called using GATK (<https://www.broadinstitute.org/gatk/>) and the SNP list was used to generate consensus sequences using SAMtools. The *PDR1* genes from all sequenced strains were aligned using MAFFT aligner (<http://www.ebi.ac.uk/Tools/msa/mafft/>) and visualized using Jalview (<http://www.jalview.org/>).
