# Supplementary figures and images for: Heteroresistance to Fluconazole Is a Continuously Distributed Phenotype among Candida glabrata Clinical Strains Associated with In Vivo Persistence
Source: mBio. 2016 Aug 2;7(4):e00655-16. doi: 10.1128/mBio.00655-16 (PMC4981708; doi:10.1128/mBio.00655-16)

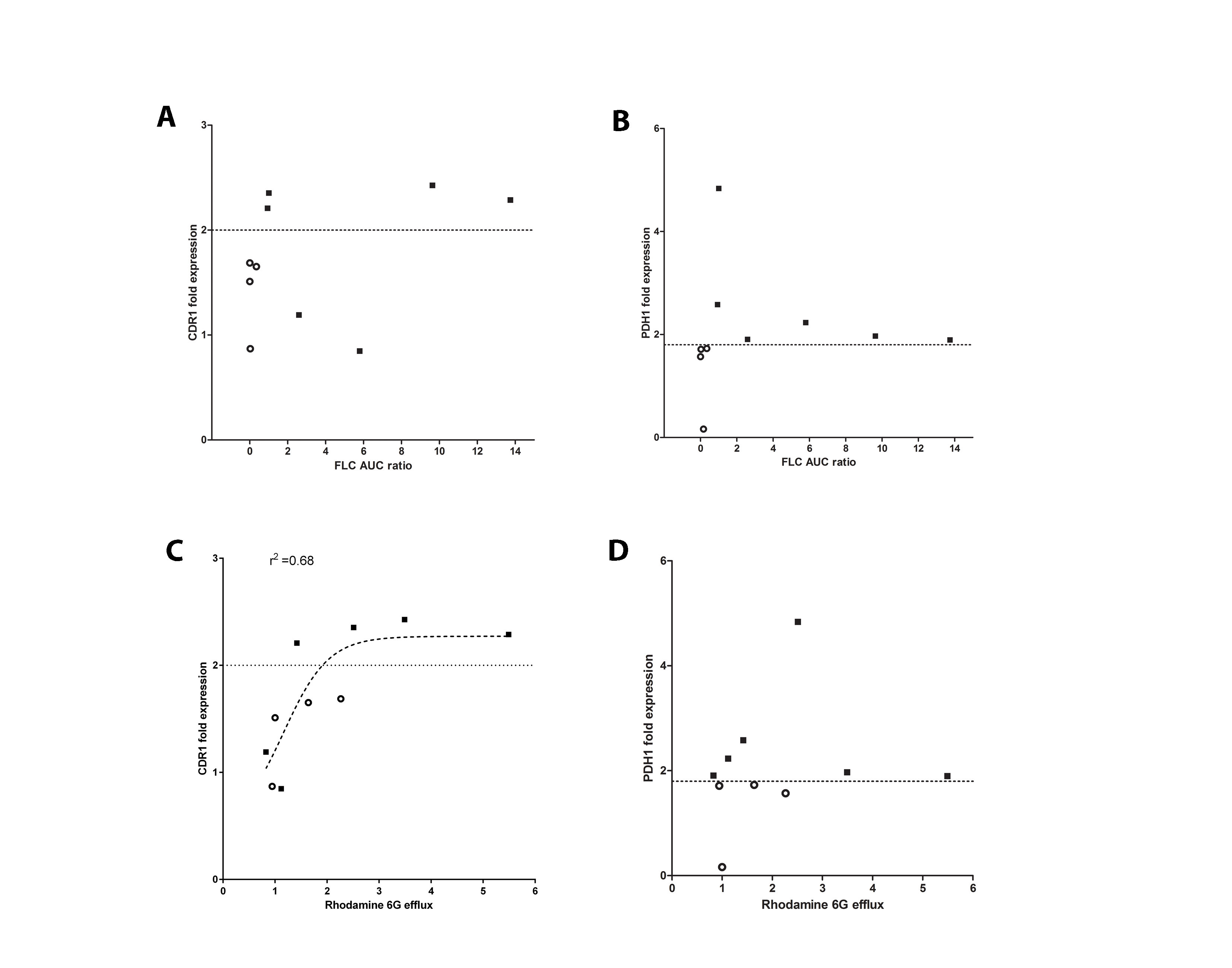

Supplement: Figure S1 — Expression of CDR1 and PDH1 is distributed bimodally and correlates with FLCHR. Scatter plots show fold expression of CDR1 (A and C) and PDH1 (B and D) as a function of the fluconazole AUCR (A and B) and rhodamine 6G efflux (C and D). Empty circles, FLCN strains; black squares, FLCHR strains. The horizontal dashed line crosses the y axis at 2 for CDR1 and 1.8 for PDH1. The graph describing CDR1 expression as a function of rhodamine 6G efflux was fitted with the equation y = ymin + [(ymax − ymin)/(1 + 10logEC50 − x)], where ymax. and ymin are the maximal and minimal gene expression values and EC50 is the value of x at the midpoint of the slope. Download [file mbo004162900sf1.tif]
