# Supplementary material for: Heteroresistance to Fluconazole Is a Continuously Distributed Phenotype among Candida glabrata Clinical Strains Associated with In Vivo Persistence
Source: mBio. 2016 Aug 2;7(4):e00655-16. doi: 10.1128/mBio.00655-16 (PMC4981708; doi:10.1128/mBio.00655-16)
Supplement: Table S1 — Clinical and reference C. glabrata strains used in this work. FLC, fluconazole; ITZ, itraconazole; VRC, voriconazole (see Materials and Methods); N, fluconazole nonheteroresistant; R, fluconazole resistant; NA, not applicable. An asterisk indicates treatment with fluconazole within 1 year prior to the isolation of C. glabrata [file mbo004162900st1.docx]

**Table S1. Clinical and reference *Candida glabrata* strains used in this work**

| ***C. glabrata* strain** | **CBS-KNAW accession number** | **GenBank accession number (Intergenic spacer CDH1-ERP6)** | **Date isolated** | **Origin** | **Fluconazole exposure*** | **MIC (mg/l)** | | | **FLC AUCR** | **FLC^HR^ phenotype** |
| --- | --- | --- | --- | --- | --- | --- | --- | --- | --- | --- |
|  |  |  |  |  |  | **FLC** | **ITZ** | **VRC** |  |  |
| TA1349 | CBS 14401 | KX271027 | 5/07/2007 | Sputum | No | 0.5 | 0.5 | 0.25 | 0.02786 | N |
| TA1399 |  | KX271019 | 11/07/2007 | Urine | No | 1 | 0.5 | 0.25 | 0.46347 | HR |
| TA2268 | CBS 14387 | KX271000 | 1/11/2007 | Urine | No | 0.5 | 0.25 | 0.06 | 9.63565 | HR |
| TA2348 |  | KX271034 | 13/11/2007 | Urine | No | 1 | 0.5 | 0.25 | 0.64099 | HR |
| TA1775 | CBS 14388 | KX271049 | 26/08/2007 | Urine | No | 0.25 | 0.25 | 0.03 | 0.16727 | N |
| TA1462 | CBS 14389 | KX271018 | 16/07/2007 | Urine | No | 1 | 0.5 | 0.25 | 5.79433 | HR |
| TA1922 | CBS 14396 | KX270999 | 5/09/2006 | Vagina | No | 2 | 0.5 | 0.125 | 2.58907 | HR |
| TA1665 |  | KX270997 | 13/08/2006 | Sputum | No | 1 | 1 | 0.5 | 0.62602 | HR |
| TA1746 | CBS 14402 | KX271050 | 16/08/2006 | Urine | No | 1 | 0.25 | 0.06 | 1.66651 | HR |
| TA1646 | CBS 14393 | KX270996 | 6/08/2007 | Other | No | 1 | 0.5 | 0.25 | 1.00000 | HR |
| TA11-304 | CBS 14398 | KX271002 | 27/9/2010 | Blood | No | 2 | 0.25 | 0.06 | 0.02593 | N |
| TA11-042 | CBS 14397 | KX271004 | 18/07/2006 | Blood | No | 2 | 0.25 | 0.25 | 0.35089 | N |
| TA11-039 |  | KX271021 | 10/07/2006 | Blood | No | 4 | 0.5 | 0.25 | 1.30638 | HR |
| TA11-016 | CBS 14390 | KX271025 | 28/02/2006 | Blood | No | 2 | 0.25 | 0.25 | 0.89844 | HR |
| TA11-012 | CBS 14391 | KX271011 | 29/01/2006 | Blood | No | 4 | 0.25 | 0.125 | 13.7433 | HR |
| TA11-005 |  | KX270994 | 28/11/2005 | Blood | No | 4 | 0.5 | 0.125 | 1.79550 | HR |
| TA11-064 | CBS 14392 | KX271003 | 28/12/2006 | Blood | Yes | 16 | 1 | 1 | 0.93386 | HR |
| TA11-056 | CBS 14395 | KX271023 | 28/09/2006 | Blood | No | 2 | 0.25 | 0.06 | 5.85433 | HR |
| TA11-092 |  | KX271026 | 14/06/2007 | Blood | No | 2 | 0.125 | 0.06 | 0.59162 | HR |
| TA11-079 | CBS 14403 |  | 14/04/2007 | Blood | No | 8 | 2 | 0.5 | 0.19266 | N |
| TA11-076 | CBS 14404 |  | 14/03/2007 | Blood | Yes | 2 | 0.25 | 0.125 | 0.22706 | N |
| TA11-078 |  | KX271014 | 16/03/2007 | Blood | No | 2 | 0.5 | 0.125 | 0.59334 | HR |
| TA11-294 |  | KX271015 | 29/08/2010 | Blood | No | 1 | 0.5 | 0.03 | 0.58302 | HR |
| TA11-276 |  | KX271006 | 20/05/2010 | Blood | No | 8 | 0.125 | 0.03 | 3.77674 | HR |
| TA11-282 | CBS 14400 | KX271029 | 13/07/2010 | Blood | No | 16 | 0.125 | 0.03 | 0.06202 | N |
| TA11-315 |  | KX271010 | 12/11/2010 | Blood | No | 2 | 0.25 | 0.06 | 8.32887 | HR |
| TA11-331 | CBS 14394 | KX271008 | 30/12/2010 | Blood | No | 2 | 0.5 | 0.06 | 0.00014 | N |
| TA11-181 | CBS 14405 | KX271012 | 02/09/2008 | Blood | No | 2 | 0.5 | 0.125 | 0.15489 | N |
| TA11-185 |  |  | 24/09/2008 | Blood | No | 2 | 0.5 | 0.06 | 0.02765 | N |
| TA11-187 |  | KX271022 | 25/09/2008 | Blood | No | 2 | 0.5 | 0.125 | 0.00012 | N |
| TA11-194 |  | KX271035 | 26/11/2008 | Blood | No | 1 | 0.125 | 0.03 | 0.10329 | N |
| TA11-201 |  |  | 12/01/2009 | Blood | No | 1 | 0.125 | 0.03 | 0.18584 | N |
| TA11-202 | CBS 14399 | KX271005 | 13/01/2009 | Blood | No | 1 | 0.125 | 0.03 | 0.57790 | HR |
| TA11-206 |  | KX271031 | 06/02/2009 | Blood | No | 1 | 0.125 | 0.03 | 0.06202 | N |
| TA11-261 |  | KX271017 | 23/01/2010 | Blood | No | 1 | 0.5 | 0.03 | 0.44376 | HR |
| TA11-264 |  | KX271024 | 29/01/2010 | Blood | No | 2 | 0.5 | 0.03 | 0.41280 | N |
| TA11-270 |  | KX271013 | 26/04/2010 | Blood | No | 1 | 0.5 | 0.03 | 0.41792 | N |
| TA11-271 |  | KX271020 | 28/04/2010 | Blood | No | 1 | 0.5 | 0.03 | 0.00012 | N |
| TA11-227 |  | KX271009 | 21/05/2009 | Blood | No | 2 | 0.25 | 0.06 | 0.12387 | N |
| TA11-217 |  | KX271036 | 11/04/2009 | Blood | No | 1 | 0.125 | 0.03 | 0.00014 | N |
| TA11-212 |  | KX271007 | 03/03/2009 | Blood | No | 1 | 0.125 | 0.03 | 0.90463 | HR |
| TA11-228 |  | KX271032 | 25/05/2009 | Blood | No | 1 | 0.25 | 0.06 | 0.08255 | N |
| TA11-255 |  | KX271016 | 24/12/2009 | Blood | No | 2 | 0.5 | 0.06 | 0.06202 | N |
| TA11-233 |  | KX271033 | 16/08/2009 | Blood | No | 1 | 0.125 | 0.03 | 0.26833 | N |
| UK1440 |  | KX271039 | 2012 | Oropharynx | Yes | 8 | 8 | 1 | 1.15059 | HR |
| UK1442 |  | KX271041 | 2012 | Blood | Yes | 8 | 8 | 1 | 3.46209 | HR |
| UK1443 |  | KX271042 | 2012 | Blood | Yes | 4 | 4 | 0.5 | 0.25804 | N |
| UK1444 |  | KX271043 | 2012 | Oropharynx | Yes | 4 | 4 | 1 | 0.42826 | N |
| UK1445 |  | KX271044 | 2012 | Blood | No | 4 | 4 | 2 | 0.77912 | HR |
| UK1446 |  | KX271045 | 2012 | Blood | No | 8 | 8 | 2 | 0.77657 | HR |
| UK1447 |  | KX271046 | 2012 | Oropharynx | Yes | 4 | 4 | 2 | 1.32601 | HR |
| UK1448 |  | KX271047 | 2012 | Urine | Yes | 4 | 4 | 2 | 1.03968 | HR |
| CBS15126 |  | KX271001 | NA | Reference | NA | 1 | 0.125 | 0.06 | 0.00014 | N |
| CBS138 |  | KX271038 | NA | Reference | NA | 8 | 0.5 | 0.25 | 0.00012 | N |
| BG2 |  | KX271037 | NA | Reference | NA | 8 | 2 | 0.5 | 0.49535 | HR |
| TA11-191 |  | KX270995 | 19/11/2008 | Blood | NA | 64 | 2 | 4 | NA | R |
| TA1708 |  | KX270998 | 13/8/2006 | Urine | NA | 64 | 2 | 2 | NA | R |
| TA11-295 |  | KX271028 | 29/8/2010 | Blood | NA | 128 | 32 | 32 | NA | R |
| TA11-326 |  | KX271030 | 10/12/2010 | Blood | NA | 64 | 4 | 2 | NA | R |
| UK1441 |  | KX271040 | 2012 | Oropharynx | Yes | 64 | 1 | 1 | NA | R |
| UK1449 |  | KX271048 | 2012 | Blood | Yes | 64 | 1 | 4 | NA | R |

FLC: fluconazole, ITZ: Itraconazole, VRC: voriconazole, FLC AUCR: fluconazole - area under the curve ratio (see Methods section), FLC^HR^: fluconazole heteroresistant phenotype, HR: fluconazole heteroresistant, N: fluconazole non-heteroresistant, R: fluconazole resistant, NA: not applicable.

* Refers to treatment with fluconazole within 1 year prior to isolation of *C. glabrata*.
