# Supplementary material for: Heteroresistance to Fluconazole Is a Continuously Distributed Phenotype among Candida glabrata Clinical Strains Associated with In Vivo Persistence
Source: mBio. 2016 Aug 2;7(4):e00655-16. doi: 10.1128/mBio.00655-16 (PMC4981708; doi:10.1128/mBio.00655-16)
Supplement: Table S2 — Nonsynonymous mutations detected in the PDR1 gene coding sequences of FLCHR and nonheteroresistant C. glabrata strains. [file mbo004162900st2.docx]

|  | **GenBank accession** | **PDR1 mutation** | | | | | | |
| --- | --- | --- | --- | --- | --- | --- | --- | --- |
|  |  | **P76S** | **I91V** | **S98L** | **P143T** | **D243N** | **I720T** | **L1107F** |
| **Fluconazole**  **heteroresistant** |  |  |  |  |  |  |  |  |
| Cg1646 | KX159459 |  |  |  |  |  |  | X |
| 11-064 | KX159460 |  |  |  |  |  |  |  |
| 11-078 | KX159462 |  |  |  |  |  |  |  |
| 11-016 | KX159463 |  |  |  |  |  |  |  |
| 11-012 | KX159465 | X |  |  | X | X |  |  |
| Cg2268 | KX159468 | X | X | X | X |  | X |  |
|  |  |  |  |  |  |  |  |  |
|  |  |  |  |  |  |  |  |  |
| **Fluconazole non-heteroresistant** |  |  |  |  |  |  |  |  |
| CBS15126 | KX159461 |  |  |  |  |  |  |  |
| Cg1775 | KX159464 |  |  |  |  |  |  |  |
| CBS138 | KX159466 | X | X | X | X |  |  |  |
| 11-304 | KX159467 | X | X | X | X |  |  |  |
|  |  |  |  |  |  |  |  |  |

**Table S2. Non-synonymous mutations detected in the coding sequence of the *PDR1* gene of fluconazole heteroresistant and non-heteroresistant *C. glabrata* strains**
